# Acute Parenchymatous Degeneration of the Kidneys in a Mare

**Published:** 1883-04

**Authors:** Frank D. Walton

**Affiliations:** Columbia Veterinary College Hospital


### V.—ACUTE PARENCHYMATOUS DEGENERATION OF THE KIDNEYS IN A MARE.

BY FRANK D. WALTON, D.V.S.

The animal was a young mare, aged 7, which had previously been well and active.

She had been at rest in the stable for several days when she was harnessed and put to light work. After being driven a short distance she became uneasy and fell into a profuse perspiration, which extended over the whole body, and it was with great difficulty that she was returned to the stable, so great was the prostration.

Dr. Wallace was first summoned to attend the patient, and found the animal quite restless, with a weak and tottering gait. An attempt was made to walk her to the hospital, but after going a block or two she lost power in the posterior extremities, and fell to the ground totally unable to raise herself.

An ambulance was summoned, and in this way she was removed to the Columbia Veterinary Hospital.

The common veterinary diagnosis azaturia was made.

The urine in this case was scanty, dark in color, and loaded with thick and tenacious mucus.

A careful examination of the urine by the pathologists of the college gave the following very positive and diagnostic results, viz.: reaction neutral, sp. gr. 1.030. It also, after filtration to get rid of the thick and tenacious mucus, responded to the various tests for albumen, namely; 1, nitric acid alone gave a marked precipitate; 2, heat and nitric acid, a marked precipitate; 3, heat and acetic acid, gave a marked precipitate, which was not dissolved by adding nitric acid, but intensified. By these tests it was positively proven that albumen was present in large quantities as well as mucus.

Sugar was also tested for, but found to be absent.

Urine, to be examined chemically, should always be filtered first.

A careful microscopic examination was made of the unfiltered portion, and it was found to contain large quantities of granular casts and granular debris, also a little pus and blood, and some of the ordinary crystalline substances commonly found in normal urine..

From these positive facts and the history of the case, the diagnosis of acute parenchymatous degeneration of the kidneys was returned.

The animal from this time on was treated accordingly. Cathartics were given to relieve the kidneys, and help in removing effete material from the blood. Hot and stimulating lotions were applied to the loins.

The mare, however, gradually grew worse, the urine more scanty. Uraemic convulsions set in, and there was a strong urinous odor from the whole body.

The following day large doses of pilocarpine and digitalis were administered, which threw the animal into a profuse perspiration, but all to no avail, for the convulsions steadily and rapidly grew worse, and passed on to coma, which caused death on the fourth day after the onset of the disease.

A necropsy was made shortly after death, and the only marked lesions was that of the kidney. The kidneys were a little enlarged, the capsule normal and non-adherent, the underlying renal tissue smooth, but pale in color. Both glands were softer than normal.

Microscopically they showed a marked granular change of the epithelium, but no special change in the interstitial tissue, the pathological changes therefore confirmed the diagnosis made prior to death.

Columbia Veterinary College Hospital, December, 1882.

[This case is one of exceedingly great interest, and we would recommend the readers to carefully peruse Dr. Porter's article upon the subject of Acute Parenchymatous Degeneration of the Kidneys.—Ed.]

